# A indicator of visceral adipose dysfunction to evaluate metabolic health in adult Chinese

**DOI:** 10.1038/srep38214

**Published:** 2016-12-01

**Authors:** Ming-Feng Xia, Ying Chen, Huan-Dong Lin, Hui Ma, Xiao-Ming Li, Qiqige Aleteng, Qian Li, Dan Wang, Yu Hu, Bai-shen Pan, Xue-Jun Li, Xiao-Ying Li, Xin Gao

**Affiliations:** 1 Department of Endocrinology and Metabolism, Zhongshan Hospital, Fudan University, Shanghai, China; 2 Institute of Chronic Metabolic Diseases, Fudan Unversity, Shanghai, China; 3 Department of Endocrinology and Metabolism, Affiliated Hospital of Nantong University, Nantong, Jiangsu, China; 4 Department of Geriatrics, Zhongshan Hospital, Fudan University, Shanghai, China; 5 Department of Laboratory Medicine, Zhongshan Hospital, Fudan University, Shanghai, China; 6 Xiamen Diabetes Institute, Department of Endocrinology and Metabolism, The First Hospital of Xiamen, Xiamen University, 55 Zhenhai Road, Xiamen 361003, China

## Abstract

Visceral adipose dysfunction is a major cause of metabolic disorders. However, there is lack of a clinical index for prediction of visceral fat dysfunction in Asians. The present study aims to establish a visceral adiposity index for evaluation of metabolic health status in Chinese, the largest Asian ethnic group. 485 subjects were recruited from Lianqian Community, Xiamen and received abdominal computed tomography(CT) for visceral fat area. A Chinese visceral adiposity index (CVAI) was created using multivariate linear regression analyses, and was further validated in 6495 subjects recruited from Changfeng Community, Shanghai. CVAI was well associated with visceral obesity (r = 0.68, P < 0.001) and HOMA-IR (r = 0.60, P < 0.001). The AUROCs were 0.89(0.88–0.90), 0.72(0.71–0.73), 0.69(0.68–0.71) and 0.67(0.65–0.68) for determination of metabolic syndrome, hypertension, diabetes and prediabetes, respectively. CVAI was more valuable compared to BMI and waist circumference in evaluation of metabolic risks (all P < 0.001), even in subjects with metabolically unhealthy normal weight (MUNW) and metabolically healthy obese/overweight (MHO). This study demonstrates that CVAI is a reliable and applicable index for evaluation of visceral fat dysfunction in Chinese. It might be used to evaluate metabolic health status in Asians.

The prevalence of obesity is rapidly rising worldwide over the past decades^1^. Since obesity increases the risk for diabetes, cardiovascular disease and cancer^2^, it becomes a major health problem and enormously increases the global health burden and health-care cost^3^,^4^.

Obesity is usually assessed by body mass index (BMI) calculated as weight in kilograms divided by height in meter squared^5^, which is well associated with type 2 diabetes, dyslipidemia and cardiovascular diseases^5^,^6^. However, BMI does not take into account the heterogeneity of body fat deposition while used as a parameter for body fat excess^7^, and fails to indentify the subgroups of metabolically healthy obese (MHO)^8^ and metabolically unhealthy normal weight (MUNW)^9^ individuals in general population. Enormous evidence demonstrates that visceral adipose tissue (VAT) rather than subcutaneous adipose tissue plays a vicious role in metabolic diseases^10^,^11^. Waist circumference is a simple parameter for abdominal adiposity and better reflects visceral obesity than BMI^12^, but waist circumference alone has limitations in distinguishing between subcutaneous and visceral fat mass^13^. Computed tomography (CT) and magnetic resonance imaging (MRI) allow quantitative measurement of VAT^14^, but they are costly and not routinely carried out in general clinical practice.

Recently, a clinical visceral adiposity index (VAI), based on waist circumference, BMI, serum triglycerides (TG), and high-density lipoprotein (HDL) cholesterol levels, was established to estimate the visceral adiposity^15^. VAI was significantly correlated with a series of metabolic diseases, including cardiovascular and cerebrovascular disease^15^, non-alcoholic fatty liver disease/NASH^16^,^17^, polycystic ovary syndrome^18^, and acromegaly^19^ in Caucasians. However, the pronounced differences in body fat distribution existed among various ethnicity^20^. Asian population is characterized by relatively higher body fat content at lower BMI values as compared with Caucasians^21^, and seems to be more prone to visceral fat accumulation^22^. A VAI for estimation of visceral fat area in Asians is definitely indispensable. In the present study, we developed and further externally validated a non-invasive clinical index to estimate visceral fat area in Chinese, the largest Asian ethnic group^23^. We investigated the performance of Chinese visceral adiposity index (CVAI) in prediction of the risk for metabolic syndrome and type 2 diabetes compared with BMI and waist circumference in Chinese population.

## Results

### Development of CVAI

To develop an index to estimate the visceral fat volume, 485 adult subjects were recruited from Lianqian Community, Xiamen, including 133 men and 352 women. The clinical characteristics of the subjects receiving CT examination for visceral fat area were shown in Supplementary Table 1. Univariate correlation analyses showed that visceral fat area was significantly associated with the parameters used in visceral adiposity index (VAI), including BMI (r = 0.49, P < 0.001), waist circumference (r = 0.56, P < 0.001), HDL cholesterol(r = −0.38, P < 0.001), and TG(r = 0.49, P < 0.001). In the setting of the Chinese visceral adiposity index (CVAI), we included the variables which are significantly associated with visceral fat area by univariate analysis. BMI, WC, serum triglyceride and HDL-C, age, smoking, alcohol drinking, serum LDL-C and total cholesterol, blood pressure, and fasting blood glucose were entered in the multivariate regression model. The combination of BMI, WC, triglyceride and HDL-c well constructed the Chinese visceral adiposity index by R^2^ of 0.40. Further inclusion of age increases R^2^ by 0.1 for female and 0.01 for male in the model, and greatly improves the model fit. While smoking, alcohol drinking, serum LDL-C, total cholesterol, blood pressure, or glucose levels only increase R^2^ by less than 0.01 and do not improve model fit significantly. Therefore, the CVAI for visceral adiposity area is estimated as follows:

Males: CVAI = −267.93 + 0.68 * age + 0.03 * BMI + 4.00 * WC + 22.00 * Log_10_TG − 16.32 * HDL-C

Females: CVAI = −187.32 + 1.71 * age + 4.23 * BMI + 1.12 * WC + 39.76 * Log_10_TG − 11.66 * HDL-C

**Figure 1:**
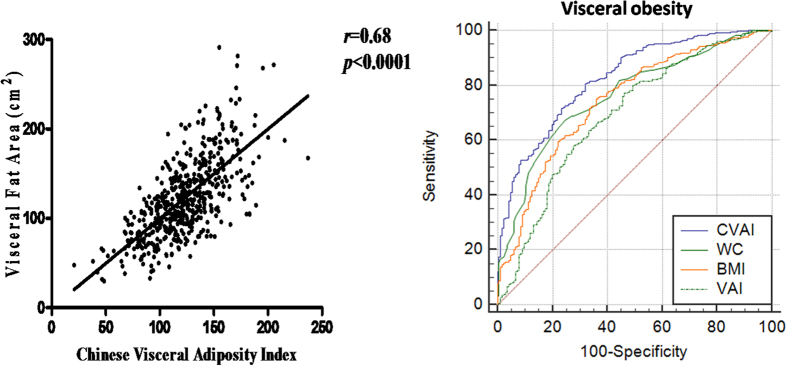
Relationship between Chinese Visceral Adiposity Index and Visceral fat area in Xiamen Lianqian population (r = 0.68, P < 0.001) (Panel on the left) and Comparison of diagnostic performance of the CVAI, BMI, waist circumference and VAI for visceral obesity (AUROC for CVAI = 0.83[0.79–0.86], P < 0.001 vs. all other parameters).

As shown in Fig. 1, CVAI was strongly and positively associated with visceral fat area (r = 0.68, P < 0.0001). The area under the ROC curve (AUROC) of CVAI for visceral obesity was 0.83(0.79–0.86) (Fig. 1), which was significantly higher than waist circumference, BMI and VAI established in Italians (AUROC: 0.69–0.76, P < 0.001) (Table 1).

### External validation of CVAI

**Figure 2:**
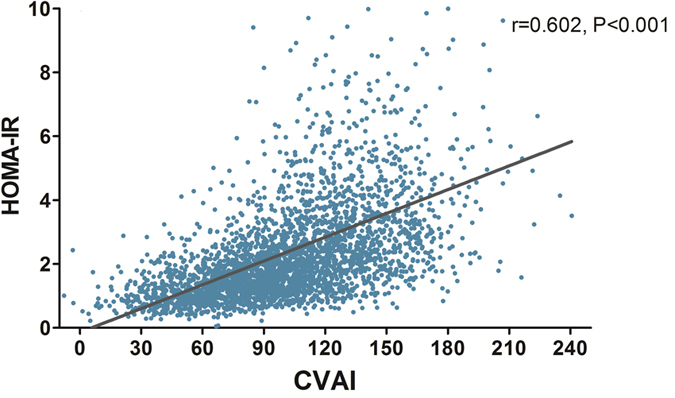
Correlation between Chinese Visceral Adiposity Index and whole-body insulin resistance level (measured by HOMA-IR) in Shanghai Changfeng Community population (r = 0.602, P < 0.001).

The CVAI was further validated in 6495 subjects (2761 males and 3734 females) from Shanghai Changfeng community. The clinical characteristics of the subjects were shown in Table 2 according to the quartiles of CVAI. As shown in Table 2, subjects with higher CVAI presented with more obese, and higher waist circumference, fasting blood glucose, blood pressure, liver enzymes and unfavourable lipid profiles (All P < 0.001). A higher proportion of cigarette smokers and alcohol drinkers was found in subjects with high CVAI. Linear correlation analysis showed that CVAI was significantly associated HOMA-IR (r = 0.602, P < 0.001) (Fig. 2).

### Diagnostic performance of CVAI

**Figure 3:**
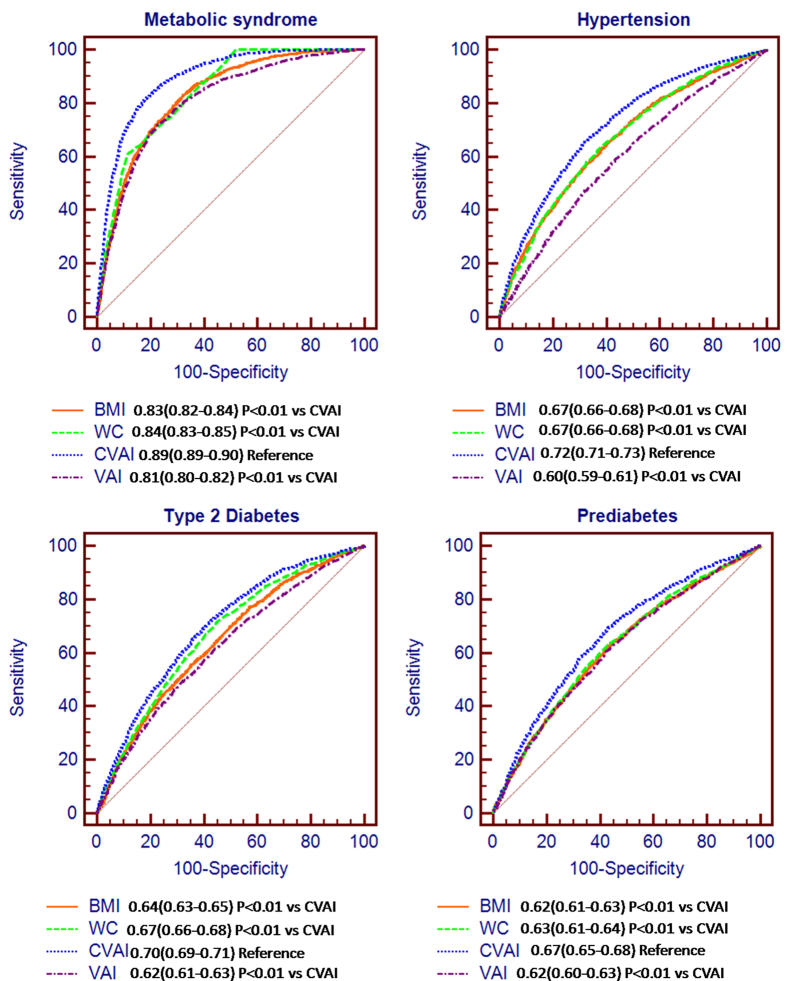
ROC curves of CVAI, BMI, waist circumference to diagnose metabolic syndrome, hypertension, diabetes and prediabetes. AUROCs were 0.89(0.88–0.90), 0.72(0.71–0.73), 0.70(0.69–0.71) and 0.67(0.65–0.68) for diagnosis of metabolic syndrome, hypertension, diabetes and prediabetes, significantly better than BMI, waist circumference and VAI established in Italians (All P < 0.001).

The AUROCs of CVAI for diagnosis of metabolic syndrome, hypertension, type 2 diabetes and prediabetes were 0.89(0.88–0.90), 0.72(0.71–0.73), 0.70(0.69–0.71) and 0.67(0.65–0.68), respectively, which is better performed than BMI, waist circumference and VAI (All P < 0.001) (Fig. 3). In the subgroup of 4871 Chinese subjects with lower visceral adiposity (CVAI at Q1-Q3 range) and an average BMI of 23 kg/m^2^, the AUROCs of CVAI for diagnosis of metabolic syndrome, hypertension, type 2 diabetes and prediabetes were 0.85(0.83–0.86), 0.68(0.67–0.69), 0.66(0.64–0.67) and 0.63(0.62–0.65), respectively (Supplementary Figure 1). Thus, the diagnostic performance of CVAI is similar in Chinese subjects with higher or lower BMI.

**Figure 4:**
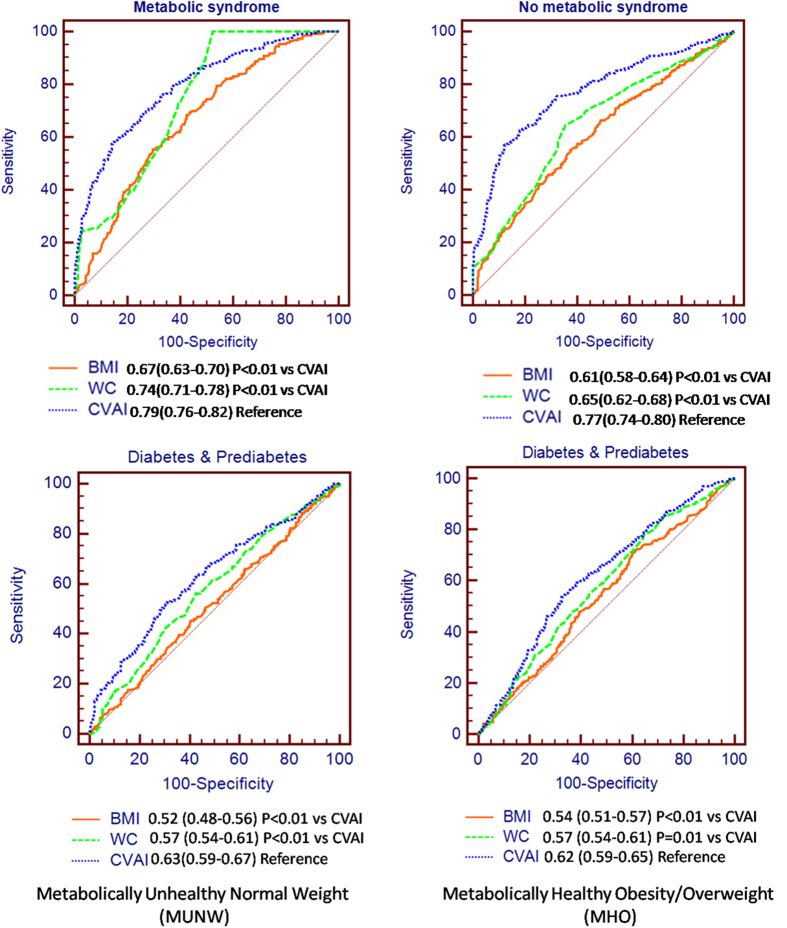
ROC curves of CVAI, BMI, waist circumference to diagnose metabolic syndrome, diabetes and prediabetes in metabolically unhealthy normal weight (panel on the left) and metabolically healthy obese/overweight subjects (panel on the right).

In subjects with MUNW and MHO/overweight, the AUROCs of CVAI were 0.79(0.76–0.82) and 0.77(0.74–0.80), respectively for diagnosing metabolic syndrome, and 0.63(0.59–0.67) and 0.62(0.59–0.65) for diabetes and prediabetes, respectively as compared with BMI and WC (AUROCs 0.61–0.74 for metabolic syndrome and 0.51–0.57 for diabetes/prediabetes) (Fig. 4).

## Discussion

In the present study, we developed a clinical index, CVAI to predict visceral adipose area in Chinese adults, which could help clinicians to screen for metabolically unhealthy individuals. CVAI is well correlated with insulin resistance and better to predict metabolic syndrome, hypertension and diabetes, particularly in MUNW and MHO subjects compared to BMI and waist circumference.

It is well known that visceral obesity plays a vicious role in insulin resistance and metabolic syndrome, a cluster of metabolic disorders, including hyperglycemia, hypertension and dyslipidemia. However, it is difficult to quantitate visceral adipose tissues using CT or MRI in large cohort studies. CVAI, a simple clinical index composed of some major parameters of metabolic syndrome, can be used to reflect visceral fat mass. A previous study showed that visceral adiposity index (VAI) could be used as an indicator of visceral adiposity and adipose tissue dysfunction to predict the risk for cardiovascular diseases, insulin resistance and metabolic syndrome in Caucasians^15^. However, VAI was poorly associated with adipose tissue area in Chinese (AUROC: 0.69[0.65–0.73], P < 0.001), possibly due to the remarkable differences in adipose tissue distribution between Caucacians and Asians. Asians could be more prone to have visceral adipose accumulation. Therefore, ethnic difference should be considered while visceral adipose area is evaluated by a clinical index.

Obesity is a heterogeneous morbidity^24^. Epidemiology studies showed that MUNW and MHO individuals accounted for a non-negligible proportion of the whole population, with an estimated prevalence ranging from 10–37% in lean and 13–29% in obese individuals^25^. In MUNW and MHO subjects, BMI can lead to miscalculation of individual metabolic risks^26^,^27^. Waist circumference, correlated with central obesity, is better to predict obesity-related metabolic abnormalities than BMI^28^. However, waist circumference is still inaccurate to distinct subcutaneous adipose tissue from the visceral fat^13^, and has poor diagnostic performance to predict prediabetes and diabetes in MUNW and MHO subjects, as shown in Fig. 4. The new index CVAI demonstrated the best performance for prediction of prediabetes and diabetes in MUNW and MHO individuals, and in whole population in our current study.

One major limitation of the current study is that the prediction value of CVAI is validated by assessing its correlation with HOMA-IR, a parameters of visceral fat function, but not directly with visceral fat areas in the validation population from Shanghai Changfeng community. Therefore, further validation of CVAI with imaging visceral fat areas in a second Chinese cohort is still needed.

In conclusion, CVAI is well correlated with visceral fat mass in Chinese adults. CVAI demonstrates a better prediction than BMI and waist circumference for metabolic disorders.

## Subjects and Methods

485 subjects (133 men and 352 women) with waist circumference greater than 90 cm for men and 85 cm for women were recruited from Lianqian Community, Xiamen to develop an index to estimate the visceral fat volume in Chinese. The detailed information about the subjects was described previously^29^,^30^, and their clinical characteristics were shown in Supplementary Table 1. All the 485 subjects received abdominal computed tomography (CT). To further validate the visceral fat index in Chinese, 6495 subjects (2761 men and 3734 women) were recruited from Changfeng Community, Shanghai^31^.

This study was approved by the Ethical Committee of the First Xiamen Hospital and the Ethical Committee of Zhongshan Hospital Fudan University, respectively. Each participant provided written informed consent. The described methods were carried out in accordance with the guidelines of the Declaration of Helsinki.

### Visceral fat area quantitation by computed tomography (CT)

Visceral fat area and subcutaneous area were measured using CT (GE Medical Systems, Milwaukee, WI, USA) at the level of fourth lumbar vertebra. Tissue compartments were measured by planimetry with a trackball-controlled cursor. The area of adipose tissue in each compartment was quantified by the program provided by the manufacture, which sums the areas of the pixels with CT values from −250 to −50 Hounsfield Units that is corresponsive to adipose tissue. Fat area was calculated by FatScan version 2.0 software (N2 System Co., Osaka, Japan). The coefficient of variation for intra-observers was <6.1% and for inter-observers was <6.6% in our current study.

### Anthropometrical and serum biochemical measurements

All participants completed a uniform questionnaire detailing history of diabetes, medication use, smoking status, and alcohol consumption. Body height and weight were measured without shoes and outer clothing. BMI was calculated according to weight in kilograms divided by square of height in meters. Waist circumference was measured using a soft tape at midway between the lowest rib and iliac crest in standing position. All blood samples were obtained after at least 12 h of fasting. Serum total cholesterol, HDL-cholesterol, triglyceride(TG) were measured using oxidase method^32^. LDL cholesterol was calculated using the Friedewald equation. Liver enzymes (ALT, AST) were measured by Ultraviolet (UV) lactate and malate dehydrogenase methods on a model 7600 automated bio-analyser (Hitachi, Tokyo, Japan). Plasma glucose concentrations were measured using glucose oxidase method. Serum insulin concentrations were determined using an electrochemiluminescence immunoassay^33^.

### Definition of visceral obesity, metabolic syndrome, diabetes, MHO and MUNW

Subjects with visceral fat area ≥ 100 cm^2^ were defined as visceral obesity^34^. According to 2006 World Health Organization (WHO), The criteria for metabolic syndrome were central obesity (waist circumference ≥ 90 cm for males and ≥80 cm for females) plus two or more of the following: antihypertensive treatment or blood pressure ≥130/85 mmHg, antidiabetic treatment or fasting blood glucose ≥5.6 mmol/L, triglycerides ≥1.7 mmol/L and HDL cholesterol <1.03 mmol/L for males and <1.29 mmol/L for females^35^. Diabetes mellitus was defined as fasting plasma glucose levels ≥7.0 mmol/L, or a 2-h post-load plasma glucose levels ≥11.1 mmol/L, or taking antidiabetic medications or insulin injection, and prediabetes was diagnosed as fasting plasma glucose 6.1–7.0 mmol/L or post-load plasma glucose 7.8–11.1 mmol/L, according to 1999 WHO criteria. HOMA- IR is calculated by dividing the product of fasting plasma glucose (mmol/L) and fasting plasma insulin (mU/L) by 22.5. Metabolic health status was defined based on insulin resistance level^36^,^37^, and HOMA-IR ≥ 2.5 was used as the cut-off value for insulin resistance in accordance with previous studies^37^. Thus, all the individuals were divided into four categories according to BMI (normal body weight: <25 kg/m^2^; obese/overweight: >25 kg/m^2^) and estimated insulin resistance level (metabolic health: insulin sensitive and HOMA-IR < 2.5; metabolic unhealth: insulin resistance and HOMA-IR ≥ 2.5)^38^,^39^. Visceral adiposity index (VAI) was calculated using the equation established in Caucasians previously^15^.

### Statistical analysis

All statistical analyses were performed using SPSS software version 15.0 (SPSS, Chicago, IL). The data were presented as mean ± SD, except for skewed variables, which were presented as the median with interquartile range (25–75%) given in parentheses. One-way analysis of variance or the Mann-Whitney U-test was used for comparisons of continuous data among groups, whereas the Chi-squared test was used for comparisons of categorical variables. Multivariate linear regression analysis was performed to establish Chinese predictive score (CVAI) for visceral fat dysfunction with candidate variables including age, BMI, waist circumference, TG, and HDL cholesterol, using visceral fat area measured by CT as reference. Receiver operating characteristic (ROC) curve analyses were used to compare the diagnostic performance of CVAI with BMI and waist circumference for risk of metabolic syndrome, and diabetes. Values of P < 0.05 were considered statistically significant for all analyses.

## Additional Information

**How to cite this article**: Xia, M.-F. *et al*. A indicator of visceral adipose dysfunction to evaluate metabolic health in adult Chinese. *Sci. Rep.* **6**, 38214; doi: 10.1038/srep38214 (2016).

**Publisher’s note:** Springer Nature remains neutral with regard to jurisdictional claims in published maps and institutional affiliations.

## Supplementary Material

Supplementary Figure

Supplementary Table

## Figures and Tables

**Table 1:** Comparison of CVAI and previously established parameters for predicting visceral obesity.

	AUC (95%CI)	P value
Chinese Visceral Adiposity Index	0.83 (0.79–0.86)	Ref.
Waist Circumference	0.76 (0.72–0.80)	0.0001
BMI	0.74 (0.70–0.78)	<0.0001
Visceral Adiposity Index (Italy)	0.69 (0.65–0.73)	<0.0001

**Table 2:** Baseline Characteristics of Shanghai Changfeng Community population according to quartiles of CVAI.

	Chinese Viseral Adipose Index				*P* Value
	Q1	Q2	Q3	Q4	
Age, y	57 (53–63)	61 (55–67)	64 (58–72)	69 (62–77)	<0.001
Gender (male/female, male%)	629/994 (38.8%)	655/969 (40.3%)	705/919 (43.4%)	772/852 (47.5%)	<0.001
Current drinker, n(%)	237 (14.6%)	259 (16.0%)	281 (17.3%)	295 (18.2%)	0.035
Current smoker, n(%)	331 (20.4%)	340 (20.9%)	401 (24.7%)	403 (24.8%)	0.001
BMI, kg/m^2^	21.1 ± 2.1	23.3 ± 2.0	25.0 ± 2.2	27.7 ± 2.8	<0.001
Waist circumference, cm	73.9 ± 5.5	80.9 ± 4.9	86.3 ± 5.1	95.1 ± 7.6	<0.001
CVAI	55.9 ± 17.3	91.4 ± 7.6	117.0 ± 7.6	154.5 ± 20.6	<0.001
SBP, mmHg	126 ± 18	133 ± 18	139 ± 18	145 ± 19	<0.001
DBP, mmHg	73 ± 10	76 ± 10	78 ± 10	78 ± 10	<0.001
Fasting blood glucose, mmol/L	4.9 (4.7–5.3)	5.2 (4.8–5.6)	5.3 (4.9–5.9)	5.6 (5.1–6.4)	<0.001
HbA1c,%	5.5 (5.3–5.7)	5.6 (5.4–5.9)	5.7 (5.4–6.0)	5.9 (5.6–6.4)	<0.001
HOMA-IR	1.2 (0.8–1.6)	1.7 (1.2–2.4)	2.2 (1.6–3.3)	3.1 (2.2–4.6)	<0.001
IGR, n(%)	227 (14.0%)	321 (19.8%)	416 (25.6%)	464 (28.6%)	<0.001
Diabetes, n(%)	124 (7.6%)	267 (16.4%)	412 (25.4%)	627 (38.6%)	<0.001
Total cholesterol, mmol/L	5.0 ± 0.9	5.1 ± 0.9	5.1 ± 0.9	5.1 ± 1.0	0.013
Triglycerides, mmol/L	0.98 (0.77–1.29)	1.32 (1.02–1.76)	1.65 (1.23–2.24)	1.91 (1.41–2.67)	<0.001
LDL-C, mmol/L	2.80 ± 0.77	2.96 ± 0.79	2.93 ± 0.82	2.86 ± 0.83	<0.001
HDL-C, mmol/L	1.71 ± 0.40	1.45 ± 0.33	1.33 ± 0.31	1.24 ± 0.28	<0.001
ALT, U/L	14 (11–19)	16 (12–21)	17 (13–24)	18 (13–26)	<0.001
AST, U/L	20 (17–23)	20 (17–23)	20 (18–24)	21 (18–25)	<0.001

## References

[b1] Ogden C. L. . Prevalence of overweight and obesity in the United States, 1999–2004 JAMA. 295, 1549–1555 (2006). 16595758 10.1001/jama.295.13.1549

[b2] Haslam D. W. & James W. P. Obesity. Lancet 366, 1197–1209 (2005). 16198769 10.1016/S0140-6736(05)67483-1

[b3] Global Burden of Metabolic Risk Factors for Chronic Diseases Collaboration (BMI Mediated Effects), . Metabolic mediators of the effects of body-mass index, overweight, and obesity on coronary heart disease and stroke: a pooled analysis of 97 prospective cohorts with 1.8 million participants. Lancet 383, 970–983 (2014). 24269108 10.1016/S0140-6736(13)61836-X PMC3959199

[b4] Huang C., Yu H. & Koplan J. P. Can China diminish its burden of non-communicable diseases and injuries by promoting health in its policies, practices, and incentives? Lancet 384, 783–792 (2014). 25176549 10.1016/S0140-6736(14)61214-9

[b5] Keys A., Fidanza F., Karvonen M. J., Kimura N. & Taylor H. L. Indices of relative weight and obesity. J Chronic Dis 25, 329–343 (1972). 4650929 10.1016/0021-9681(72)90027-6

[b6] Colditz G. A., Willett W. C., Rotnitzky A. & Manson J. E. Weight gain as a risk factor for clinical diabetes mellitus in women. Ann Intern Med 122, 481–486 (1995). 7872581 10.7326/0003-4819-122-7-199504010-00001

[b7] Despres J. P., Lemieux I. & Prud′homme D. Treatment of obesity: need to focus on high risk abdominally obese patients. BMJ 322, 716–720 (2001). 11264213 10.1136/bmj.322.7288.716 PMC1119905

[b8] Rey-López J. P. . The prevalence of metabolically healthy obesity: a systematic review and critical evaluation of the definitions used. Obes Rev. 15, 781–790 (2014). 25040597 10.1111/obr.12198

[b9] St-Onge M. P., Janssen I. & Heymsfield S. B. Metabolic syndrome in normal-weight Americans: new definition of the metabolically obese, normal-weight individual. Diabetes Care 27, 2222–2228 (2004). 15333488 10.2337/diacare.27.9.2222

[b10] Despres J. P. . Role of deep abdominal fat in the association between regional adipose tissue distribution and glucose tolerance in obese women. Diabetes 38, 304–309 (1989). 2645187 10.2337/diab.38.3.304

[b11] Hwang Y. C. . Visceral abdominal fat accumulation predicts the conversion of metabolically healthy obese subjects to an unhealthy phenotype. Int J Obes (Lond). 39, 1365–1370 (2015). 25920773 10.1038/ijo.2015.75 PMC4564328

[b12] Nazare J. A. . Usefulness of measuring both body mass index and waist circumference for the estimation of visceral adiposity and related cardiometabolic risk profile (from the INSPIRE ME IAA study). Am J Cardiol. 115, 307–315 (2015). 25499404 10.1016/j.amjcard.2014.10.039

[b13] Pouliot M. C. . Waist circumference and abdominal saggital diameter: best simple anthropometric indices of abdominal visceral adipose tissue accumulation and related cardiovascular risk in men and women. Am J Cardiol 73, 460–468 (1994). 8141087 10.1016/0002-9149(94)90676-9

[b14] Graffy P. M. & Pickhardt P. J. Quantification of hepatic and visceral fat by CT and MR imaging: relevance to the obesity epidemic, metabolic syndrome and NAFLD. Br J Radiol. 20151024 (2016). 26876880 10.1259/bjr.20151024 PMC5258166

[b15] Amato M. C. . Visceral Adiposity Index: a reliable indicator of visceral fat function associated with cardiometabolic risk. Diabetes care 33, 920–922 (2010). 20067971 10.2337/dc09-1825 PMC2845052

[b16] Musso G., Cassader M. & Gambino R. Diagnostic accuracy of adipose insulin resistance index and visceral adiposity index for progressive liver histology and cardiovascular risk in nonalcoholic fatty liver disease. Hepatology 56, 788–789 (2012). 22371327 10.1002/hep.25677

[b17] Petta S. . Visceral adiposity index is associated with significant fibrosis in patients with non-alcoholic fatty liver disease. Aliment Pharmacol Ther. 35, 238–247 (2012). 22117531 10.1111/j.1365-2036.2011.04929.x

[b18] Amato M. C., Verghi M., Galluzzo A. & Giordano C. The oligomenorrhoic phenotypes of polycystic ovary syndrome are characterized by a high visceral adiposity index: a likely condition of cardiometabolic risk. Hum Reprod. 26, 1486–1494 (2011). 21447694 10.1093/humrep/der088

[b19] Ciresi A., Amato M. C., Pizzolanti G. & Giordano Galluzzo C. Visceral adiposity index is associated with insulin sensitivity and adipocytokine levels in newly diagnosed acromegalic patients. J Clin Endocrinol Metab. 97, 2907–2915 (2012). 22679062 10.1210/jc.2012-1518

[b20] Deurenberg P., Yap M. & van Staveren W. A. Body mass index and percent body fat: a meta analysis among different ethnic groups. Int J Obes Relat Metab Disord 22, 1164–1171 (1998). 9877251 10.1038/sj.ijo.0800741

[b21] Deurenberg P., Deurenberg-Yap M. & Guricci S. Asians are different from Caucasians and from each other in their body mass index/body fat per cent relationship. Obes Rev 3, 141–146 (2002). 12164465 10.1046/j.1467-789x.2002.00065.x

[b22] Camhi S. M. . The relationship of waist circumference and BMI to visceral, subcutaneous, and total body fat: sex and race differences. Obesity 19, 402–408 (2011). 20948514 10.1038/oby.2010.248 PMC3960785

[b23] Etler D. A. Recent developments in the study of human biology in China: a review. Hum Biol. 64, 567–585 (1992). 1644424

[b24] Tchernof A. & Després J. P. Pathophysiology of human visceral obesity: an update. Physiol Rev. 93, 359–404 (2013). 23303913 10.1152/physrev.00033.2011

[b25] Badoud F., Perreault M., Zulyniak M. A. & Mutch D. M. Molecular insights into the role of white adipose tissue in metabolically unhealthy normal weight and metabolically healthy obese individuals. FASEB J. 29, 748–758 (2015). 25411437 10.1096/fj.14-263913

[b26] Ruderman N., Chisholm D., Pi-Sunyer X. & Schneider S. The metabolically obese, normalweight individual revisited. Diabetes 47, 699–713 (1998). 9588440 10.2337/diabetes.47.5.699

[b27] Karelis A. D., St-Pierre D. H., Conus F., Rabasa-Lhoret R. & Poehlman E. T. Metabolic and body composition factors in subgroups of obesity: what do we know? J Clin Endocrinol Metab 89, 2569–2575 (2004). 15181025 10.1210/jc.2004-0165

[b28] Zimmet P. . The metabolic syndrome: a global public health problem and a new definition. J Atheroscler Thromb 12, 295–300 (2005). 16394610 10.5551/jat.12.295

[b29] Zhang H. J. . Irisin is inversely associated with intrahepatic triglyceride contents in obese adults. J Hepatol. 59, 557–62 (2013). 23665283 10.1016/j.jhep.2013.04.030

[b30] Zhang H. J. . Effects of Moderate and Vigorous Exercise on Nonalcoholic Fatty Liver Disease: A Randomized Clinical Trial. JAMA Intern Med. 176, 1074–1082 (2016). 27379904 10.1001/jamainternmed.2016.3202

[b31] Gao X. . The Shanghai Changfeng Study: a community-based prospective cohort study of chronic diseases among middle-aged and elderly: objectives and design. Eur J Epidemiol. 25, 885–893 (2010). 21120588 10.1007/s10654-010-9525-6

[b32] Li X. . Liver fat content is associated with increased carotid atherosclerosis in a Chinese middle-aged and elderly population: the Shanghai Changfeng study. Atherosclerosis. 224, 480–485 (2012). 22884916 10.1016/j.atherosclerosis.2012.07.002

[b33] Bian H. . Increased liver fat content and unfavorable glucose profiles in subjects without diabetes. Diabetes Technol Ther. 13, 149–155 (2011). 21284482 10.1089/dia.2010.0101

[b34] Examination Committee of Criteria for ‘Obesity Disease′in Japan & Japan Society for the Study of Obesity. New criteria for ‘obesity disease′in Japan. Circ J. 66, 987–992 (2002). 12419927 10.1253/circj.66.987

[b35] Alberti K. G., Zimmet P. & Shaw J. Metabolic syndrome: A new worldwide definition. A consensus statement from the International Diabetes Federation. Diabet. Med. 23, 469–480 (2006). 16681555 10.1111/j.1464-5491.2006.01858.x

[b36] Durward C. M., Hartman T. J. & Nickols-Richardson S. M. All-cause mortality risk of metabolically healthy obese individuals in NHANES III. J Obes. 2012, 460321 (2012). 23304462 10.1155/2012/460321 PMC3523154

[b37] Stefan N., Häring H. U., Hu F. B. & Schulze M. B. Metabolically healthy obesity: epidemiology, mechanisms, and clinical implications. Lancet Diabetes Endocrinol. 1, 152–162 (2013). 24622321 10.1016/S2213-8587(13)70062-7

[b38] Calori G. . Prevalence, metabolic features, and prognosis of metabolically healthy obese Italian individuals: the Cremona Study. Diabetes Care. 34, 210–215 (2011). 20937689 10.2337/dc10-0665 PMC3005463

[b39] Guy-Marino Hinnouho . Metabolically Healthy Obesity and Risk of Mortality: Does the definition of metabolic health matter? Diabetes Care 36, 2294–2300 (2013). 23637352 10.2337/dc12-1654 PMC3714476

