# Supplementary material for: A indicator of visceral adipose dysfunction to evaluate metabolic health in adult Chinese
Source: Sci Rep. 2016 Dec 1;6:38214. doi: 10.1038/srep38214 (PMC5131270; doi:10.1038/srep38214)
Supplement: Supplementary Figure [file srep38214-s1.pdf]

A indicator of visceral adipose dysfunction to evaluate metabolic health in adult Chinese

Ming-Feng Xia[a,b*], Ying Chen[c*], Huan-Dong Lin[a,b], Hui Ma[d], Xiao-Ming Li[a,b], Qiqige Aleteng[a,b], Qian Li[a,b], Dan Wang[a,b], Yu Hu[d], Bai-shen Pan[e], Xue-Jun Li[f], Xiao-Ying Li [a,b$], Xin Gao[a,b$]

[a]*Department of Endocrinology and Metabolism, Zhongshan Hospital, Fudan University, Shanghai, China*

[b]*Institute of Chronic Metabolic Diseases, Fudan Unversity, Shanghai, China*

cDepartment of Endocrinology and Metabolism, Affiliated Hospital of Nantong University, Nantong, Jiangsu, China.

[d]*Department of Geriatrics, Zhongshan Hospital, Fudan University, Shanghai, China*

[e]*Department of Laboratory Medicine, Zhongshan Hospital, Fudan University, Shanghai, China*

[f] *Xiamen Diabetes Institute, Department of Endocrinology and Metabolism, The First Hospital of Xiamen, Xiamen University, 55 Zhenhai Road, Xiamen 361003, China.*

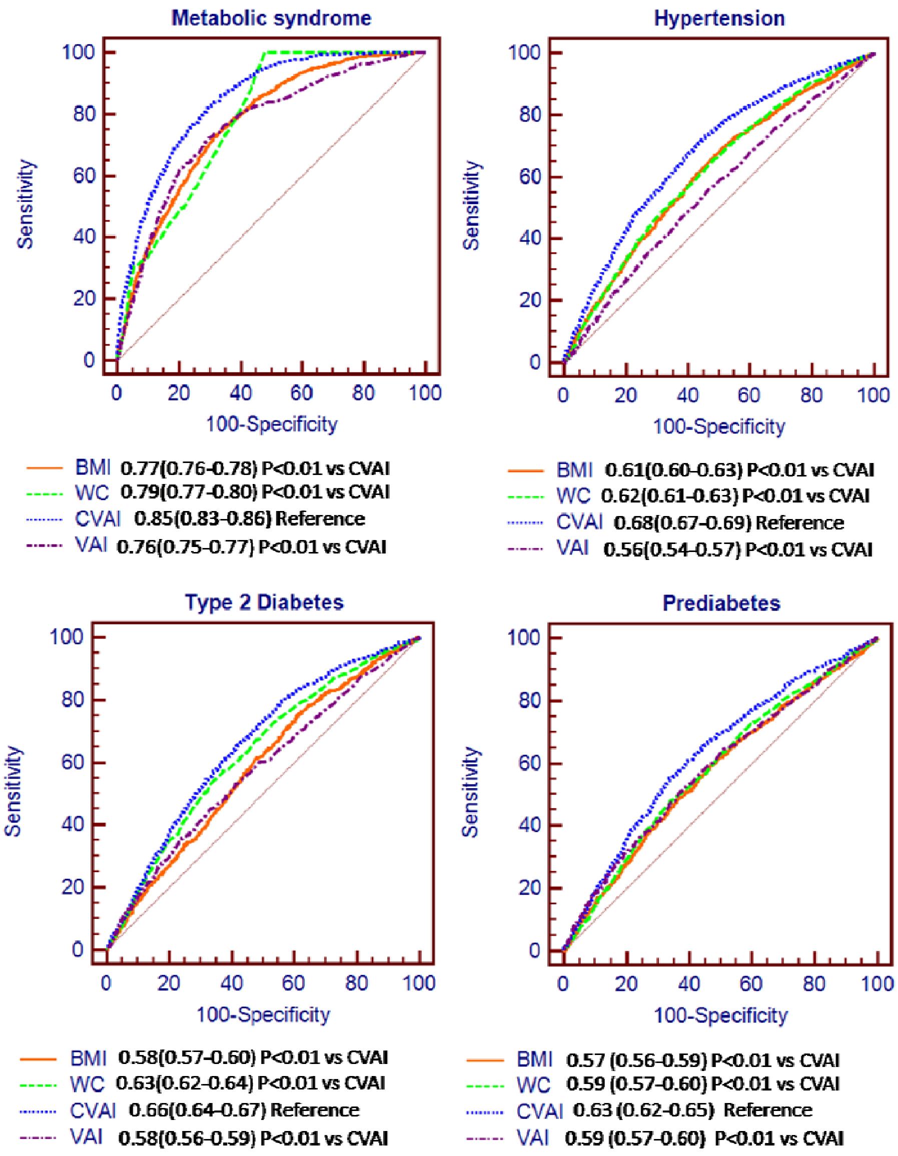

Supplementary Figure 1. ROC curves of CVAI, BMI, waist circumference to diagnose metabolic syndrome, hypertension, diabetes and prediabetes in the subgroup of Chinese with lower visceral adiposity (CVAI at Q1-Q3 range). AUROCs were 0.85(0.83-0.86), 0.68(0.67-0.69), 0.66(0.64-0.67) and 0.63(0.62-0.65) for diagnosis of metabolic syndrome, hypertension, diabetes and prediabetes, significantly better than BMI， waist circumference and VAI established in Italians (All $P<0.001$).
