# Supplementary material for: A indicator of visceral adipose dysfunction to evaluate metabolic health in adult Chinese
Source: Sci Rep. 2016 Dec 1;6:38214. doi: 10.1038/srep38214 (PMC5131270; doi:10.1038/srep38214)
Supplement: Supplementary Table [file srep38214-s2.doc]

**A indicator of visceral adipose dysfunction to evaluate metabolic health in adult Chinese**

Ming-Feng Xia^a,b*^, Ying Chen^c*^, Huan-Dong Lin^a,b^, Hui Ma^d^, Xiao-Ming Li^a,b^, Qiqige Aleteng^a,b^, Qian Li^a,b^, Dan Wang^a,b^, Yu Hu^d^, Bai-shen Pan^e^, Xue-Jun Li^f^, Xiao-Ying Li ^a,b$^, Xin Gao^a,b$^

^a^*Department of Endocrinology and Metabolism, Zhongshan Hospital, Fudan University, Shanghai, China*

^b^*Institute of Chronic Metabolic Diseases, Fudan Unversity, Shanghai, China*

*cDepartment of Endocrinology and Metabolism, Affiliated Hospital of Nantong University, Nantong, Jiangsu, China.*

^d^*Department of Geriatrics, Zhongshan Hospital, Fudan University, Shanghai, China*

^e^*Department of Laboratory Medicine, Zhongshan Hospital, Fudan University, Shanghai, China*

^f^ *Xiamen Diabetes Institute, Department of Endocrinology and Metabolism, The First Hospital of Xiamen, Xiamen University, 55 Zhenhai Road, Xiamen 361003, China.*

| Table S1 Baseline Characteristics of 485 participants from Lianqian community | | |
| --- | --- | --- |
|  | Male  N=133 | Female  N=352 |
| Age, y | 53.4±7.7 | 54.3±6.9 |
| BMI , kg/m2 | 27.7±2.4 | 27.0±3.1 |
| Waist circumference, cm | 97.0±5.4 | 93.0±6.8 |
| Visceral fat area, cm^2^ | 147.4±48.4 | 114.8±37.8 |
| Liver fat content, % | 16.6±11.1 | 12.9±9.5 |
| SBP, mmHg | 134.0±14.5 | 127.8±16.4 |
| DBP, mmHg | 81.5±9.6 | 76.3±9.9 |
| Fasting blood glucose, mmol/L | 5.55±0.50 | 5.51±0.52 |
| 2h-postload blood glucose,mmol/L | 7.50±1.95 | 7.99±1.92 |
| Triglycerides, mmol/L | 2.28±1.47 | 1.83±1.49 |
| Total cholesterol, mmol/L | 5.72±0.93 | 5.77±1.02 |
| LDL cholesterol, mmol/L | 3.76±0.91 | 3.76±1.01 |
| HDL cholesterol, mmol/L | 1.14±0.19 | 1.37±0.25 |
| HOMA-IR | 2.81 (2.09, 4.04) | 2.85 (1.85, 4.01) |
